# The Rolf Method of Structural Integration on Fascial Tissue Stiffness, Elasticity, and Superficial Blood Perfusion in Healthy Individuals: The Prospective, Interventional Study

**DOI:** 10.3389/fphys.2020.01062

**Published:** 2020-09-15

**Authors:** Grzegorz Jędrzejewski, Martyna Kasper-Jędrzejewska, Paweł Dolibog, Renata Szyguła, Robert Schleip, Tomasz Halski

**Affiliations:** ^1^Faculty of Health Sciences, University of Opole, Opole, Poland; ^2^Department of Sport and Health Sciences, Technical University Munich, Munich, Germany; ^3^Diploma University of Applied Sciences, Bad Sooden-Allendorf, Germany

**Keywords:** structural integration, fascial tissue, superficial blood perfusion, stiffness, elasticity

## Abstract

**Introduction**: There are multiple theories surrounding the physiological impact of structural integration (SI) with little evidence or research corroborating any of these. The aim of the study was to assess the effectiveness of 10 sessions of SI on fascial tissue (FT) superficial blood perfusion, stiffness, and elasticity in 13 healthy women.

**Methods**: This was a prospective, interventional study. The primary outcome measures were FTs’ superficial blood perfusion, stiffness, and elasticity of bilateral selected FT points on the body. Data were collected before and after 10 sessions of SI intervention. Statistical analysis was performed using the non-parametric Wilcoxon test (intragroup comparison).

**Results**: The superficial blood perfusion increased significantly in the most selected FT points on the body (*p* < 0.05). SI interventions produced significant decreases in selected points (brachioradialis, biceps brachii, and trapezius; *p* < 0.05) of FT stiffness and significant increases in elasticity (brachioradialis, biceps brachii, triceps surae, and trapezius; *p* < 0.05), especially in the FT of the right (dominant) upper limb.

**Conclusion**: A 10-session of SI demonstrated positive effects on increasing superficial blood perfusion contributed to a decrease in FT stiffness and an increase in elasticity properties in the dominant upper limb. Data collection for this study is currently underway, and the trial is registered at ISRCTN.com with the identifier: ISRCTN46707309.

## Introduction

Structural integration (SI) is a holistic form of manual therapy that was developed by Ida Rolf, which consists of fascial therapies combined with motor re-education (Baur et al., 2017). Its main purpose is to restore the functional capabilities of the human body (Jacobson, 2011) by influencing its biomechanics in the gravity field (Jacobson, 2011; Jacobson et al., 2015; Baur et al., 2017). The phenomenon of biotensegrity plays a key role in SI therapy, in which the skeletal system functions due to dynamic changes in the muscular, fascial, and nervous systems. In a biomechanically well-functioning body, the forces acting on it are evenly distributed, dispersed, and transmitted, thanks to a network of viscoelastic tension elements (Dischiavi et al., 2018). It can certainly be concluded that SI acts mechanically on sensitive receptors in the soft tissues, which is likely to result in changes in their structure and function (Loghmani et al., 2017). Mechanotherapy can be defined as any manual intervention using mechanical stimuli to influence biological changes in tissues through mechanotransduction processes with the aim of improving their function (Loghmani and Whitted, 2016). Mechanotransduction, in turn, is the mechanism by which specialized cells (e.g., telocytes and fibroblast) change the mechanical stimulus (twisting, tension, compression, stretching, bending, and friction) applied during manual intervention into chemical activity (Chaitow, 2018a). Endoscopic fascia studies conducted by Guimberteau confirm the appearance of mechanotransduction during manual interventions involving the manipulation of soft tissues (Guimberteau and Armstrong, 2015).

The principles of SI, for many years, have been based on a holistic approach to patients’ reported muscular and biomechanical dysfunctions. The functional definition of the fascia, proposed in 2019, can help explain these etiologies in a theoretical way (Schleip et al., 2019b). Studies by Horton (2015) have shown that fascial tissue (FT) is innervated by nociceptors and mechanoreceptors, which supply information to both the somatic and autonomic nervous systems. It contains smooth muscle-like cells with a myofibroblast cellular structure, which can change its shape in response to mechanical stimuli (Horton, 2015) and is also richly vascularized (Behm and Wilke, 2019). Fascial therapy, apart from relieving pain and improving the range of joint mobility, can have a direct effect on molecular pathways, cellular response, and the structure and function of tissues as well as their healing, repair, and regeneration (Best et al., 2013; Costello et al., 2016; Kojidi et al., 2016). There is also some evidence that when performed on musculoskeletal and myofascial chains, therapy can induce long-lasting effects on tissues, which can manifest as changes in their strength and range of motion (Dischiavi et al., 2018).

The ability of soft tissues to recover their shape after deformation caused by strain is called their elasticity – the higher the elasticity, the easier the tissue returns to its original shape. Moreover, elasticity plays an important role in the mechanism of proper muscle contraction (Herzog, 2019). On the other hand, stiffness determines resistance to external force and is one of the basic indicators of energy storage in a muscle-tendon unit, which effectively controls its movement (Kocur et al., 2019). According to Findley (2012), the stiffness and elasticity of FTs play an important role in human ballistic movements, and changes in them may manifest themselves in the form of various disorders of the musculoskeletal system (e.g., chronic pain or susceptibility to injury), as well as reduced muscle strength and coordination disorders (Wilke et al., 2018b; Chen et al., 2019; Schleip et al., 2019a). In addition, reduced hydration of FTs reduces their elasticity, leading to a kind of gluing (tissue adhesion) and the formation of painful trigger points (Behm and Wilke, 2019). Trigger points may affect the formation of musculoskeletal and fascial imbalance, which is biomechanically related to the persistence of abnormal posture and motor dysfunction (Kisilewicz et al., 2018; Wilke et al., 2018a; Behm and Wilke, 2019).

A review of internet medical databases (PubMed, PEDro, Web of Science, EBSCO) indicates a small number of reports about SI therapy (Silverman et al., 1973; Cottingham et al., 1988a,b; Jones, 2004; James et al., 2009; Jacobson, 2011; Hansen et al., 2014; Jacobson et al., 2015; Loi et al., 2015; Baur et al., 2017). In the Physiotherapy Evidence Database (PEDro) of randomized trials, systematic reviews, and clinical practice guidelines in physiotherapy (Yamato et al., 2017), there are two items of relatively high methodological value in PEDro scale with 6/10 score (Loi et al., 2015) and 7/10 score (Jacobson et al., 2015) and one of lower value with 4/10 score (Cottingham et al., 1988b). The total PEDro score is gained by counting the number of “yes” responses for items 2–11 (item 1 is not used for calculation of the total PEDro score) and ranges from 0 to 10 points (Yamato et al., 2017). In 2011, Jacobson (2011) published a review of 12 preliminary, small sample clinical studies, single case, and small case series, in which SI is evaluated in terms of, among other things, the effects on the nervous system, self-esteem, anxiety reduction, gait function, musculoskeletal pain, muscle tone, body balance, and the range of motion. A detailed analysis of 12 studies allowed the author to conclude that the clinical efficacy and mechanisms of the influences of SI on the human body are limited by the small number of experimental groups and the lack of control groups across studies (Jacobson, 2011). Moreover, due to the increasing use of SI in the reduction of pain and musculoskeletal disorders, further research is justified (Jacobson et al., 2015; Tarsha et al., 2020). Improvement of professional qualifications based on the latest research findings is a necessary condition, with evidence-based physiotherapy (EBP) becoming increasingly desirable. The world confederation for physical therapy defines EBP as a commitment to use in practice the best available scientific evidence to inform decisions concerning the choice of diagnostic methods and the most effective methods of therapy or prevention (Scurlock-Evans et al., 2014; Veras et al., 2016).

The aim of this study was to use quantitative methods to investigate changes in select FT parameters including stiffness, elasticity, and superficial blood perfusion after SI intervention. We hypothesized that 10 sessions of SI would produce a decrease in the stiffness and an increase in elasticity and superficial blood perfusion of the selected FT points. To the best of our knowledge, the presented findings are the first to show the effects of SI on FT using modern, objective, and non-invasive measurement tools.

## Materials and Methods

### Design

This was a prospective, interventional study evaluating the persistent changes in specific superficial blood perfusion, stiffness, and elasticity parameters after 10 SI sessions. The study period (recruitment, data collection, and intervention) was December 2019 to March 2020 in the Clinical Research Laboratory in the Physiotherapy Department of the Opole Medical School, Poland. The study was approved by the independent Bioethics Committee of the Opole Medical School, Poland nr KB/205/FI2019. Upon enrollment, participants gave written informed consent after a thorough explanation of the procedures involved, and being told that their anonymity would be preserved and that they could leave the study at any time. The research project was prospectively registered as an interventional trial in the ISRCTN registry (registration no. ISRCTN46707309). This project is supported by partial financing from the grant within the budget of Opole Medical School, agreement no. 63/ROP/CRUZ/2018.

### Participants

Fifteen participants were qualified and recruited through self-selection from Opole Medical School based on the inclusion (age between 20 and 25 years, consent to participate in the study) and exclusion criteria (pregnancy, nursing period, and non-specific neuromuscular disease) for the study. Two potential participants were excluded because they did not meet the inclusion criteria or declined to participate. Finally, the study group consisted of 13 generally healthy women (all of them were non-smokers, two had non-regular menstrual cycle, and two used contraceptive pills) at the age of 23.38 ± 0.5 years.

### Structural Integration Intervention Protocol

A trained SI therapist with 3 years of practical experience conducted the SI interventions. Each intervention lasted for 60 min and patients underwent a total of 10 sessions (one session/week) at the Clinical Research Laboratory in the Physiotherapy Department of the Opole Medical School, Poland. SI therapy was performed in the following order (Jacobson, 2011; see Supplementary Table 1).

### FT Superficial Blood Perfusion, Stiffness, and Elasticity Measurements

Measurements of superficial blood perfusion, stiffness, and elasticity of selected FT were conducted before first SI session, after which each participant underwent a 10-session of SI intervention/once a week. About 72 h after the 10th session, the participants were given another stiffness, elasticity, and superficial blood perfusion examination. The participants were informed about the procedure and asked to stay as relaxed as possible during each measurement. All parameters were measured on both sides (bi-laterally) and at the same FT points (Table 1). FT point location and the methods of their determination were selected based on the publications of various authors (Pruyn et al., 2016; Kisilewicz et al., 2018; Weber et al., 2020). In the absence of a precise description of the measurement point determination, the present study suggests the location of specific anthropometric points, which were treated as reference for determining the measurement point. The choice of measuring points was based on the image of the whole body subjected to SI.

**Table 1:** Description of measurement fascial tissue (FT) point location in participants’ supine, lateral, and prone positions.

Fascial tissue/muscle	Position	Description of measurement point location
Brachioradialis (BR)	Supine	Forearm in intermediate position. The measurement point was between the lateral epicondyle of the humerus and the styloid process of the radial bone – 5 cm below the lateral epicondylitis, on the previously marked line.
Biceps brachii, caput longum (BB)	Supine	Forearm in supine position. The measurement point was between line connecting the medial epicondyle of the humerus and acromion – 7 cm above the medial epicondyle, on a previously determined line.
Adductors (ADD)	Lateral	The measurement point was between line connecting base of patella and ischial tuberosity – 20 cm above the ischial tuberosity.
Triceps surae (TS)-medial head	Prone	Feet hang freely behind the table. We determine the line connecting the calcaneus with the medial epicondyle of the femoral bone. We measured 10 cm below the epicondyle on the previously designated line.
Erector spinae (ES)	Prone	3 cm laterally from the L3 spinous process. Forehead based on the dorsal side of the hand.
Trapezius (T)	Prone	3 cm from the Th3 spinous process. Forehead based on the dorsal side of the hand.

Superficial blood perfusion measurements were taken using the laser speckle contrast analysis (LASCA) technique (see Supplementary Figure 1). The area covered by the measurement had the shape of an ellipse with a field of 2 cm/1.5 cm and was made at a distance of 10 cm from the selected point. The results were given in perfusion units (PUs). The perfusion value given in conventional units is linearly dependent on the average blood cell velocity, and non-linearly, on their concentration (Cutolo et al., 2018). Calibration of the system was performed 1 day prior to the experiment. To minimize the risk of confounding factors, daylight and other sources of light were diminished as well as movements of the device during the measurements. The participants were instructed to breathe normally and not to talk during the measurements.

The IndentoPRO Tissue Compliance Meter is a handheld, non-invasive device that evaluates mechanical characteristics of selected FTs, which is useful for the assessment of their function and metabolic efficiency (see Supplementary Figure 2). The device was developed by the Fascia Research Group from Ulm University in cooperation with the Department of Human Movement Sciences at the University of Chemnitz, Germany (Chaitow, 2018b; Gonzalez et al., 2018). To ensure conformity between the diverse assessments, the localization was marked with a permanent marker and the penetration depth was set at 5 mm and was chosen based on previous preparatory examinations with a similar patient group (*n* = 8, data not published).

For each of the FTs, three stiffness and five elasticity measurements (with 1 s pauses between them) were performed, which were averaged for analysis. In order to minimize human error, a series of three measurements was taken for each muscle and fascial structure (10 min interval between each series) and their results were averaged. The results for stiffness were given in N/mm and for elasticity as % difference between the most extreme measured values during the measurement.

### Data Analysis

Statistical analyses were performed using the STATISTICA software 13.3 (StatSoft, Inc., version 13, United States). The values of the measurements were compared using the non-parametric Wilcoxon test. A significance level of *p* < 0.05 was considered to be statistically significant.

## Results

### Comparative Analysis of the Superficial Blood Perfusion Results

Table 2 shows the intragroup analysis of the superficial blood perfusion results (PU) including the comparison between the pre‐ and post-SI intervention results. The superficial blood perfusion values in the post-SI intervention results were higher in all the FTs measured, with significance at the following measurement points: BR-L (*p* = 0.001), BR-R (*p* = 0.002), BB-L (*p* = 0.023), BB-R (*p* = 0.016), TS-L (*p* = 0.001), TS-R (*p* = 0.005), ES-L (*p* = 0.013), ES-R (*p* = 0.033), and T-R (*p* = 0.013). In the remaining points, the increase in blood perfusion was statistically insignificant. For ADD-R (*p* = 0.075) and T-L (*p* = 0.075), the increase was at the limit of statistical significance (Table 2). These changes are also clearly apparent when presented as the mean percentage difference from pre‐ to post-SI intervention (Table 2 and Supplementary Figure 3).

**Table 2:** The intragroup analysis of the superficial blood perfusion results.

Fascial tissue/muscle	Pre-SI intervention Mean ± SD (PU)	Post-SI intervention Mean ± SD (PU)	Relative percentage change in perfusion	*p* value
BR-L	4.92 ± 4.35	10.54 ± 5.35	209.16	0.001^*^
BR-R	9.83 ± 4.27	17.56 ± 6.48	104.79	0.002^*^
BB-L	19.64 ± 8.08	24.79 ± 9.49	36.40	0.023^*^
BB-R	25.86 ± 8.13	33.64 ± 10.42	35.07	0.016^*^
ADD-L	41.57 ± 11.57	48.30 ± 9.36	22.94	0.116
ADD-R	44.07 ± 10.20	54.85 ± 15.90	31.72	0.075
TS-L	15.72 ± 6.54	25.34 ± 8.77	96.69	0.001^*^
TS-R	19.51 ± 6.17	25.63 ± 8.90	37.59	0.005^*^
ES-L	34.50 ± 8.11	41.48 ± 8.77	23.47	0.013^*^
ES-R	37.47 ± 8.32	43.69 ± 10.00	19.12	0.033^*^
T-L	34.98 ± 6.58	40.29 ± 8.52	17.74	0.075
T-R	34.67 ± 6.22	41.44 ± 10.00	20.38	0.013^*^

BR-L and BR-R, left and right musculus brachio-radialis; BB-L and BB-R, left and right musculus biceps-brachii; ADD-L and ADD-R, left and right adductores; TS-L and TS-R, left and right musculus triceps surae; ES-L and ES-R, left and right musculus erector spinae; T-L and T-R, left and right musculus trapezius.

* *and bold values means: statistical significant p < 0.05*.

### Comparative Analysis of the Stiffness and Elasticity Results

Table 3 shows the intragroup analysis of stiffness including comparison between pre‐ and post‐ SI intervention results. The stiffness values in post-SI intervention results were substantially lower at the following points: BR-R (*p* = 0.028), BB-R (*p* = 0.025), and T-R (*p* = 0.012). It should be emphasized that in all participants these changes concerned the FTs of the dominant upper limb. The rest of the values were statistically insignificant (Table 3 and Supplementary Figures 4, 5).

**Table 3:** The intragroup analysis of the stiffness results.

Fascial tissue/muscle	Pre-SI intervention Mean ± SD (N/mm)	Post-SI intervention Mean ± SD (N/mm)	Relative percentage change in stiffness ± SD	*p* value
BR-L	1.20 ± 0.30	1.23 ± 0.22	5.18	0.576
BR-R	**1.33 ± 0.35**	**1.12 ± 0.20**	−12.83	0.028^*^
BB-L	0.73 ± 0.22	0.75 ± 0.15	11.51	0.442
BB-R	**0.90 ± 0.30**	**0.68 ± 0.19**	−18.15	0.025^*^
ADD-L	0.49 ± 0.10	0.50 ± 0.06	5.64	0.442
ADD-R	0.48 ± 0.10	0.47 ± 0.09	−1.06	0.142
TS-L	0.69 ± 0.18	0.69 ± 0.11	5.93	0.937
TS-R	0.66 ± 0.16	0.74 ± 0.11	14.84	0.059
ES-L	0.56 ± 0.19	0.56 ± 0.18	2.81	0.972
ES-R	0.53 ± 0.19	0.54 ± 0.16	5.71	0.972
T-L	0.84 ± 0.24	0.77 ± 0.18	−4.39	0.637
T-R	**0.85 ± 0.25**	**0.69 ± 0.19**	−16.65	0.012^*^

BR-L and BR-R, left and right musculus brachio-radialis; BB-L and BB-R, left and right musculus biceps-brachii; ADD-L and ADD-R, left and right adductores; TS-L and TS-R, left and right musculus triceps surae; ES-L and ES-R, left and right musculus erector spinae; T-L and T-R, left and right musculus trapezius.

* *and bold values means: statistical significant p < 0.05*.

Table 4 shows the intragroup analysis of elasticity including comparison between pre‐ and post-SI intervention results. It was observed that there was an increase in the elasticity of the assessed FTs at all measured points except ADD-R and TS-R. The elasticity values in the post-SI intervention results were substantially higher at the following points: BR-R (*p* = 0.016), BB-R (*p* = 0.011), and T-L (*p* = 0.043), and lower in TS-R (*p* = 0.043). In the case of BR-R and BB-R, with a simultaneous, statistically significant decrease in stiffness, a significant increase in elasticity was noted. The rest of the values were statistically insignificant (Table 4). See the Supplementary Material for graph with mean percentage for the three parameters (stiffness, elasticity, and superficial blood perfusion).

**Table 4:** The intragroup analysis of the elasticity results.

Fascial tissue/muscle	Pre-SI intervention Mean ± SD (%)	Post-SI intervention Mean ± SD (%)	Relative percentage change in elasticity	*p* value
BR-L	8.25 ± 4.59	8.15 ± 2.02	24.69	0.875
BR-R	**6.15 ± 2.50**	**9.03 ± 2.44**	67.52	0.016^*^
BB-L	10.86 ± 5.63	13.31 ± 4.45	50.26	0.196
BB-R	**8.17 ± 4.92**	**16.87 ± 10.89**	162.94	0.011^*^
ADD-L	19.26 ± 11.17	18.00 ± 7.67	27.36	0.972
ADD-R	21.68 ± 9.13	19.40 ± 11.09	−5.60	0.388
TS-L	11.50 ± 5.79	10.59 ± 3.54	9.77	0.638
TS-R	**11.75 ± 5.69**	**8.12 ± 3.66**	−23.68	0.043^*^
ES-L	10.72 ± 5.22	13.76 ± 3.46	48.39	0.055
ES-R	12.91 ± 8.60	12.69 ± 5.32	20.23	0.279
T-L	**9.72 ± 4.98**	**13.98 ± 6.06**	66.02	0.043^*^
T-R	14.41 ± 16.90	14.61 ± 6.26	56.30	0.133

BR-L and BR-R, left and right musculus brachio-radialis; BB-L and BB-R, left and right musculus biceps-brachii; ADD-L and ADD-R, left and right adductores; TS-L and TS-R, left and right musculus triceps surae; ES-L and ES-R, left and right musculus erector spinae; T-L and T-R, left and right musculus trapezius.

* *and bold values means: statistical significant p < 0.05*.

## Discussion

The main aim of this study was an objective assessment of the influence of 10 sessions of SI on FT stiffness, elasticity, and superficial blood perfusion in young, healthy women. The results indicate that a 10-session SI intervention produced significantly greater increases in superficial blood perfusion at selected FTs points. Moreover, the analysis of the results of pre‐ and post-SI intervention showed an improvement in elasticity and a decrease in stiffness parameters. This study is the first to compare changes in FT stiffness, elasticity and superficial blood perfusion following holistic and specified SI intervention.

The potential to increase blood perfusion by various kind of manual therapy techniques was assessed by researchers and clinicians, but the results have been controversial and inconclusive (Sefton et al., 2010; Portillo-Soto et al., 2014; Nelson, 2015; Plakornkul et al., 2016). In a study on the effectiveness of myofascial manual techniques in improving the functioning of the circulatory system, works by Ramos-Gonzales and Shah must be mentioned, despite their use of other measuring tools. Ramos-González et al. (2012) conducted a randomized intervention study in 65 postmenopausal women with stage I or II venous insufficiency. In the study, myofascial release therapy (MRT) was administered for 30 min daily, five times a week, for 8 weeks. The control and experimental group patients underwent physical venous return therapy for 10 weeks, during which the experimental group also received 20 sessions of MRT. Venous perfusion velocity was determined with a venous Doppler probe and was found to be significantly higher (*p* < 0.048) in the experimental group than in the control group after the 10-week treatment programs (Ramos-González et al., 2012). A study by Shah et al. (2017) showed that a single, 30-min myofascial treatment session led to improved blood perfusion in 44 healthy participants, which is also consistent with the results of the blood perfusion measurements conducted in this study. The researchers used near infrared spectroscopy and demonstrated that myofascial therapy increases peripheral blood flow at the paraspinal muscles in healthy participants compared to Kinesio Taping and sham TENS (control group; Shah et al., 2017).

Our study utilized the non-invasive LASCA technique to measure peripheral blood perfusion, which has been widely used to monitor these parameters within muscles as it has a higher spatial and temporal resolution than other non-invasive tools used to dynamically evaluate blood perfusion (Cutolo et al., 2018; Ruaro et al., 2018). A speckle pattern is produced by interference light reflected from different parts of the illuminated surface (Hohenauer et al., 2019). To our knowledge, this study was the first to investigate the effects of SI intervention on FTs blood perfusion using LASCA. The major effects of SI found in this study were markedly higher levels of blood perfusion at most selected FTs examined in the study. A possible explanation for the observed increase in blood perfusion may be due to the restoration of normal tissue structure and function following SI. According to a report by Schleip (2003a), stimulation of Ruffini corpuscles during fascial therapy has a “fascial release” effect in SI called melting, which is achieved by stimulation of intrafascial mechanosensory nerve endings. This stimulation (apart from local tissue relaxation) probably results in activation of the parasympathetic system, reduced capillary constriction and improved blood perfusion (Schleip, 2003a). Moreover, myofascial techniques used in SI may influence the ground substance of the soft tissues and mechanoreceptors, contributing to decreased excessive muscle tension, changes in local fluid dynamics, capillary constriction, and improved local blood perfusion (Schleip, 2003a,b). Moreover, changes in fluid dynamics could be explained by the last study of Menon et al. (2020), which using proton T1ρ relaxation mapping to determine intramuscular glycosaminoglycan (GAG) content. Their data suggest that the application of the fascial manipulation (FM) method seems to decrease the concentration of unbound water inside the deep fascia in the patients with chronic elbow pain and stimulate to produce the correct quality and quantity of hyaluronan (HA). HA is considered a lubricant that allows the gliding i.e., between individual layers of the deep fascia, and its high concentration can increase the viscosity in the extracellular matrix (ECM) of the FT, generating pain. Results shows that FM seems to hydrolyze the overflow amount of self-aggregate GAG/HA reducing the viscoelasticity of the ECM in the affected elbow regions after treatment (Menon et al., 2020). It is also proposed that SI may have an effect on myofascial adhesions, which could theoretically increase blood perfusion (Portillo-Soto et al., 2014). In healthy FTs, blood perfusion consists of an organized network of capillaries, arterioles, and venules. Increasing blood perfusion through this network either through physical exercise or through clinical treatment provides a greater amount of oxygen to muscles, affecting their structure to yield potentially higher levels of physical performance (Caliskan et al., 2019).

The increases in blood perfusion that we observed in our study as a result of SI may contribute to improved muscle stiffness *via* antidromic or local vasodilation (Sato-Suzuki et al., 2019). Concerning fascial stiffness and elasticity parameters, we noticed significant reductions in stiffness and increased elasticity. Work by Barassi et al. (2019) has also shown the effectiveness of myofascial manual techniques in improving the stiffness and elasticity of FT, which is consistent with the results of our study. Barassi et al. evaluated the effect of neuromuscular manual and vibration therapy on elasticity, stiffness, and musculoskeletal tonus measured with a Myoton Pro. The project involved 60 women aged 50–70 years who reported urinary incontinence in their gynecological histories. Each participant underwent vibration therapy (300 Hz for 15 min) in the abdomen, pelvic floor, lumbar spine, and gluteus maximus. Specific manual techniques were then performed around the diaphragm, lumbar, piriformis, and quadratus lumborum muscles, as well as sacrotuberous and sacrospinous ligaments. It was found that parameters related to muscle stiffness and tonus were lowered, while those related to the flexibility of specific fascial structures increased. The observed changes were significant both in the right and left side comparisons as well as before and after eight interventions (Barassi et al., 2019).

The IndentoPRO used in this study is a novel clinical tool used to assess fascial biomechanical properties (i.e., stiffness and elasticity), and no studies, to date, have assessed the reliability of this tool in measuring select FTs after 10 sessions of SI, which makes it difficult to compare results. The IndentoPRO is an improved version of the tissue compliance meter, originally introduced by Fischer in 1987 and which has been shown to yield a high intra‐ and inter-tester reliability (Wernicke et al., 2009). It seems that knowledge of FT stiffness and elasticity will allow for a better understanding of, and provide further evidence on the local effects of holistic SI therapy. According to Schleip et al. (2019a), short-term decreases in FT stiffness might be able to impact nervous system coordination. For example, increased fascial stiffness due to altered sympathetic nervous system activation may modify proprioception and coordination, which could possibly contribute to injury (Schleip et al., 2019a). Another explanation for the reduction of the stiffness of FTs may be the restoration of their shiftability to each other and re-hydration in response to applied mechanical stimuli (Zügel et al., 2018; Harper et al., 2019). Moreover, Stecco et al. (2013, 2018) suggest that myofascial release modifies the expression of HA in fascia, which then results in a lower viscosity in the ground substance and also in increased gliding ability.

An increase in FT elasticity, a major positive outcome observed in this study, is liable to bring about muscle strengthening, which is needed to increase control over the body in terms of functions such as physical performance (Barassi et al., 2019). According to Klingler et al., increased fascial stiffness has been reported in ulnar nerve compression syndrome and in myofascial neck pain (Schleip and Klingler, 2019). If responses to SI therapy result in a reduction of tissue stiffness in dominant upper limb, as found in this study, it can be hypothesized that the therapy could lead to a reduction in pain and restoration of lost functions in patients with muscular disorders. This is the team’s plan in the continuation of this research, particularly given that currently, fascial therapy is an area of great interest as it may prove to be more effective than standard physical therapy (Harper et al., 2019). Other study suggests that the dominant upper limb tends to be stiffer than the non-dominant one, which may support the argument that daily activities and specific physical activity have an impact on tissue properties (Durand et al., 2019). Moreover, the stiffer tissue on the dominant side might lead to a higher risk of pain compared to the non-dominant side (Zhang et al., 2019). Significant decrease of stiffness and increase of elasticity in the dominant upper limb in this study may be the result of the state of these tissues “before therapy”. It is worth to mention that various studies demonstrated the effect of the menstrual cycle and sex hormones on increasing FT elasticity (Lee and Petrofsky, 2018; Fede et al., 2019; Vita et al., 2019). Therefore, researchers should be aware of these physiological effects in young healthy women, which are mentioned in the limitations of the study.

### Limitations of the Study

This study investigated the effects of SI in healthy women and should attempt to make a comparison between different myofascial techniques or with massage methods in symptomatic participants. The depth, pressure, and speed of SI techniques could not be fully standardized in the study; however, in attempts to standardize these variables, the same therapist was used to conduct all the SI interventions. The authors plan to extend these studies by adding randomized control groups as “sham SI intervention” as well as participants reporting pain. The results reported will consistent with the consolidated standards of reporting trials (CONSORT) guidelines. Moreover, the limitation of this study is the lack of information about menstrual cycle and women’s sex hormones, and its effects on FT. In continued studies by our team, such evaluation will be included. Data collection for this trial is currently underway and the trial is registered at ISRCTN.com with the identifier: ISRCTN46707309. Enrollment is expected to continue through 2020 with long-term outcomes completed by 2025 for all participants.

## Conclusion

A 10-session of SI intervention demonstrated positive effects on increasing superficial blood perfusion in the selected FT points. In addition, SI intervention contributed to a decrease in FT stiffness and an increase in elasticity properties in the dominant upper limb. To assess clinical parameters in the long-term, additional studies are required.

## Data Availability Statement

The datasets generated and analyzed for this study can be obtained by contacting the corresponding author (MK-J).

## Ethics Statement

The studies involving human participants were reviewed and approved by Bioethics Committee of the Opole Medical School, Poland nr KB/205/FI2019. The patients/participants provided their written informed consent to participate in this study.

## Author Contributions

GJ and TH conceived and planned the study. GJ, MK-J, and RSz carried out the experiment. PD and GJ contributed to the data curation and interpretation of the results. GJ, MK-J, and RSz took the lead in writing the manuscript. RS and TH supervised the project. All authors discussed the results, contributed to manuscript revision, read and approved the submitted version.

### Conflict of Interest

The authors declare that the research was conducted in the absence of any commercial or financial relationships that could be construed as a potential conflict of interest.
