# Supplementary material for: The Rolf Method of Structural Integration on Fascial Tissue Stiffness, Elasticity, and Superficial Blood Perfusion in Healthy Individuals: The Prospective, Interventional Study
Source: Front Physiol. 2020 Sep 15;11:1062. doi: 10.3389/fphys.2020.01062 (PMC7522439; doi:10.3389/fphys.2020.01062)
Supplement: Supplementary file 1 [file Data_Sheet_1.pdf]

Supplementary Material

Table 1. Description of ten Structural Integration intervention (Jacobson 2011).

Session	Intervention
**1**	Increase length and pliability of FT on anterior aspect of trunk, allowing freer respiratory movement of ribs, of FT connecting shoulder girdle to rib cage and hips to pelvis.
**2**	Increase consistency of FT pliability in feet, ankles and knees, increasing the support they provide for the upper body.
**3**	Increase anterior–posterior and cephalic–caudal pliability in FT of the lateral side of the body and in left/right and anterior/posterior balance, increase independence of thorax from pelvis.
**4**	Increase pliability, left/right and anterior/posterior balance of FT of the medial aspect of legs and pelvic floor.
**5**	Increase pliability and left/right and surface to deep balance in FT comprising the anterior aspect of the pelvis and lumbar spine.
**6**	Increase pliability and left/right and surface to deep balance in FT comprising posterior aspect from heel to back.
**7**	Increase pliability and left/right and anterior/posterior balance in FT of the cranium and cervical spine.
**8**	Increase FT pliability and left/right balance in the hands, wrists, elbows, and arms; increase biomechanical flow between upper extremities and spine.
**9**	Increase FT pliability comprising the lower extremities through hips and pelvis; increase biomechanical flow between lower extremities and spine.
**10**	Optimize biomechanical flow through extremities, shoulder, and pelvic girdles to spine; increase overall uniformity of tonus.

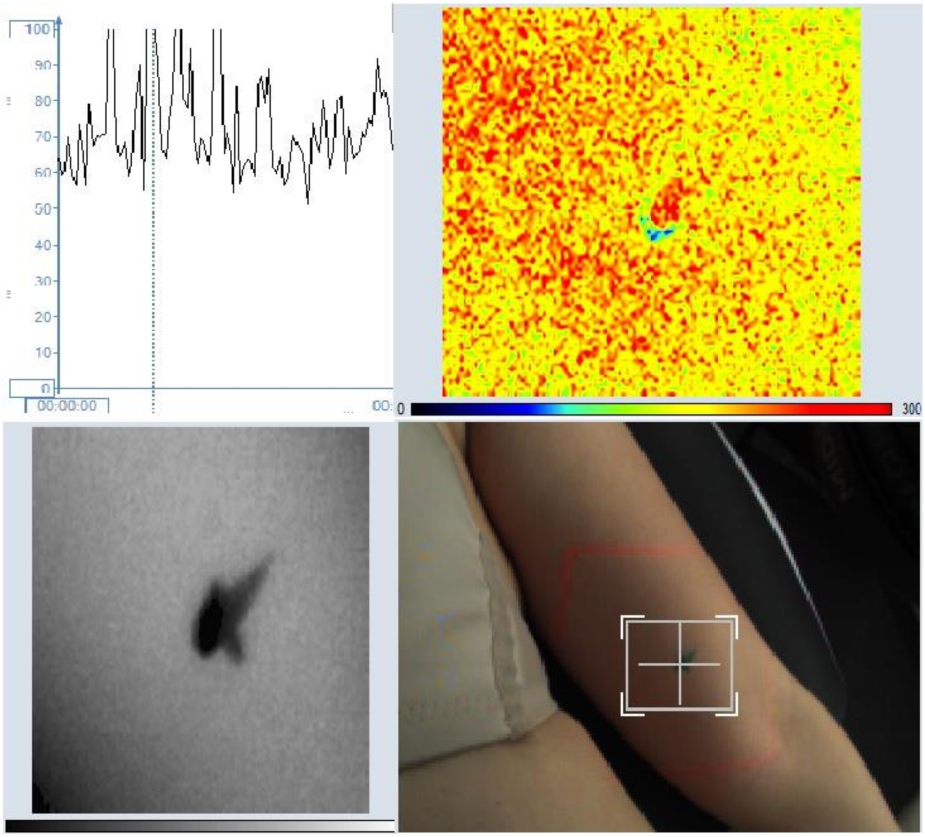

Figure 1. Measurement over the Biceps Brachii, caput longum (BB) point location by Laser speckle contrast analysis 146 (LASCA) technique (blood perfusion).

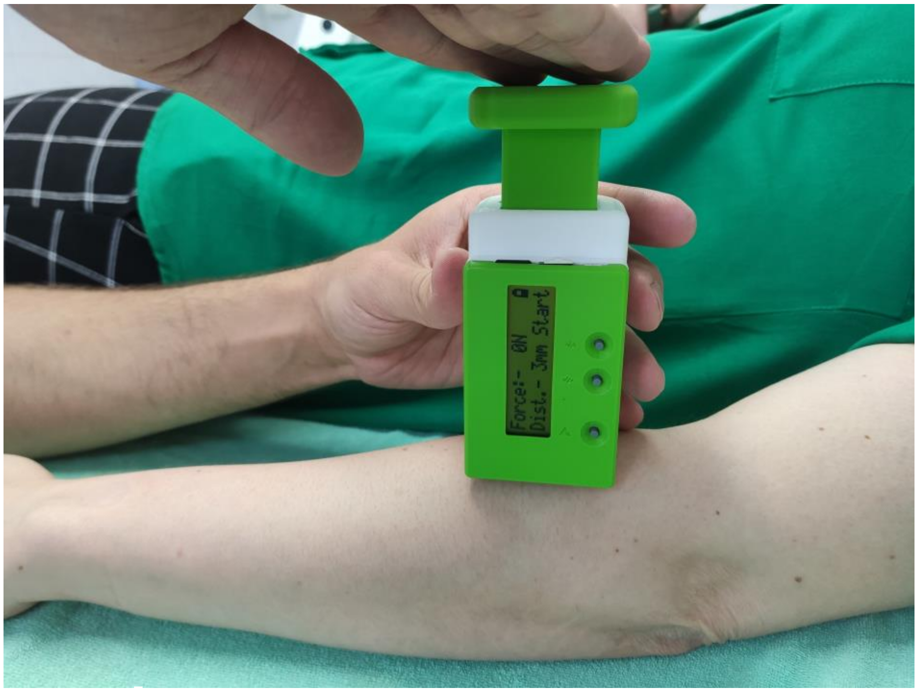

Figure 2. Measurement over the Brachioradialis (BR) point location by IndentoPRO Tissue Compliance meter (stiffness and elasticity).

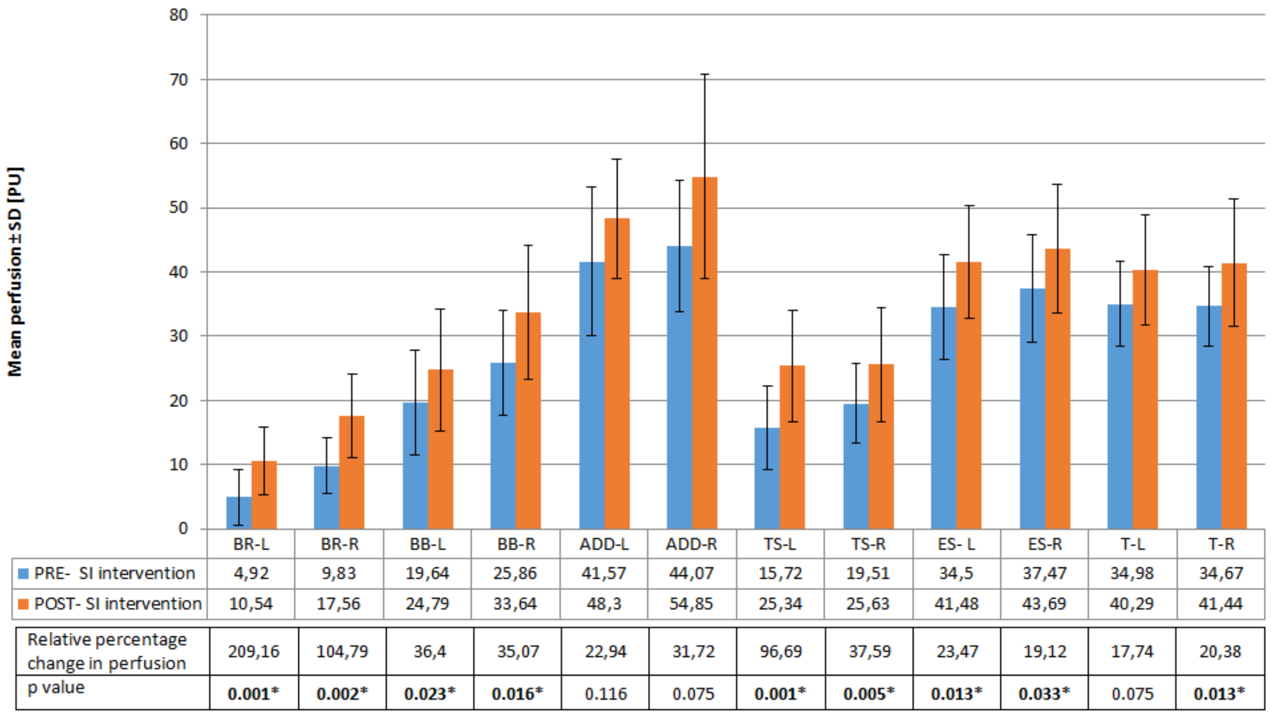

	BR-L	BR-R	BB-L	BB-R	ADD-L	ADD-R	TS-L	TS-R	ES- L	ES-R	T-L	T-R
PRE- SI intervention	4,92	9,83	19,64	25,86	41,57	44,07	15,72	19,51	34,5	37,47	34,98	34,67
POST- SI intervention	10,54	17,56	24,79	33,64	48,3	54,85	25,34	25,63	41,48	43,69	40,29	41,44
Relative percentage change in perfusion	209,16	104,79	36,4	35,07	22,94	31,72	96,69	37,59	23,47	19,12	17,74	20,38
p value	**0.001***	**0.002***	**0.023***	**0.016***	0.116	0.075	**0.001***	**0.005***	**0.013***	**0.033***	0.075	**0.013***

Figure 3. Mean change in FT blood perfusion pre and post SI intervention.

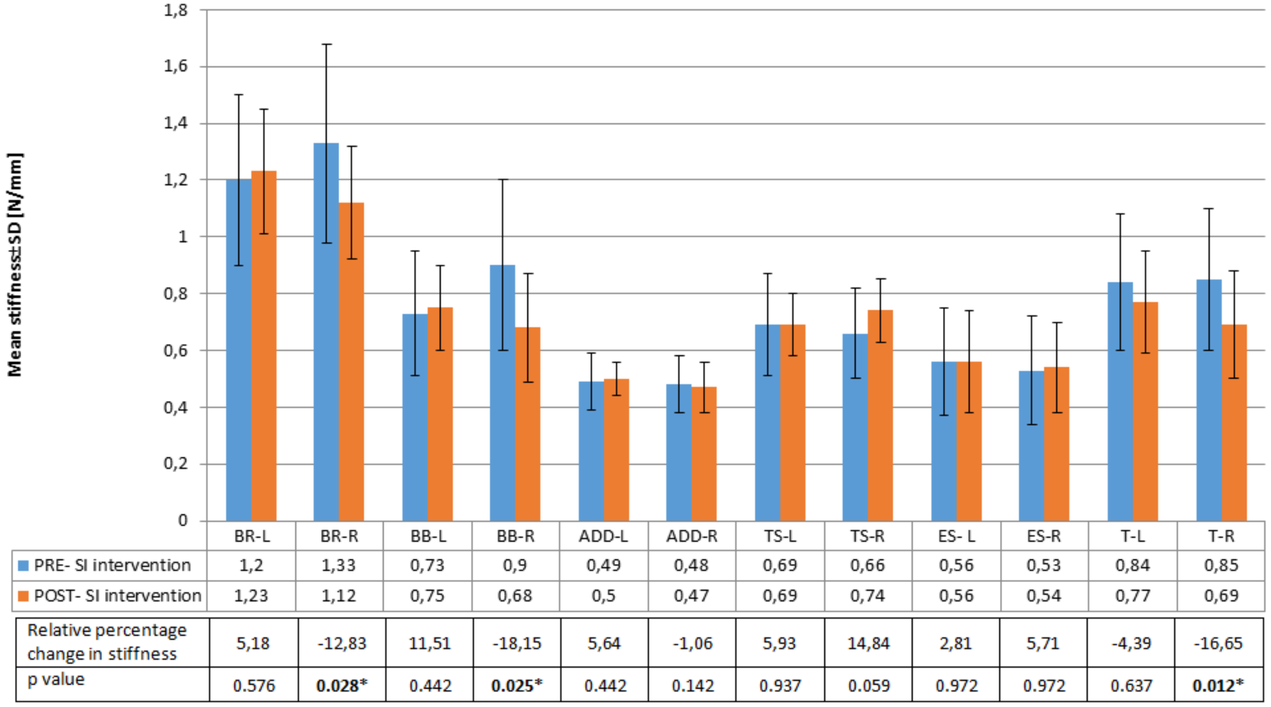

	BR-L	BR-R	BB-L	BB-R	ADD-L	ADD-R	TS-L	TS-R	ES- L	ES-R	T-L	T-R
PRE- SI intervention	1,2	1,33	0,73	0,9	0,49	0,48	0,69	0,66	0,56	0,53	0,84	0,85
POST- SI intervention	1,23	1,12	0,75	0,68	0,5	0,47	0,69	0,74	0,56	0,54	0,77	0,69
Relative percentage change in stiffness	5,18	-12,83	11,51	-18,15	5,64	-1,06	5,93	14,84	2,81	5,71	-4,39	-16,65
p value	0.576	**0.028***	0.442	**0.025***	0.442	0.142	0.937	0.059	0.972	0.972	0.637	**0.012***

Figure 4. Mean change in FT stiffness pre and post SI intervention.

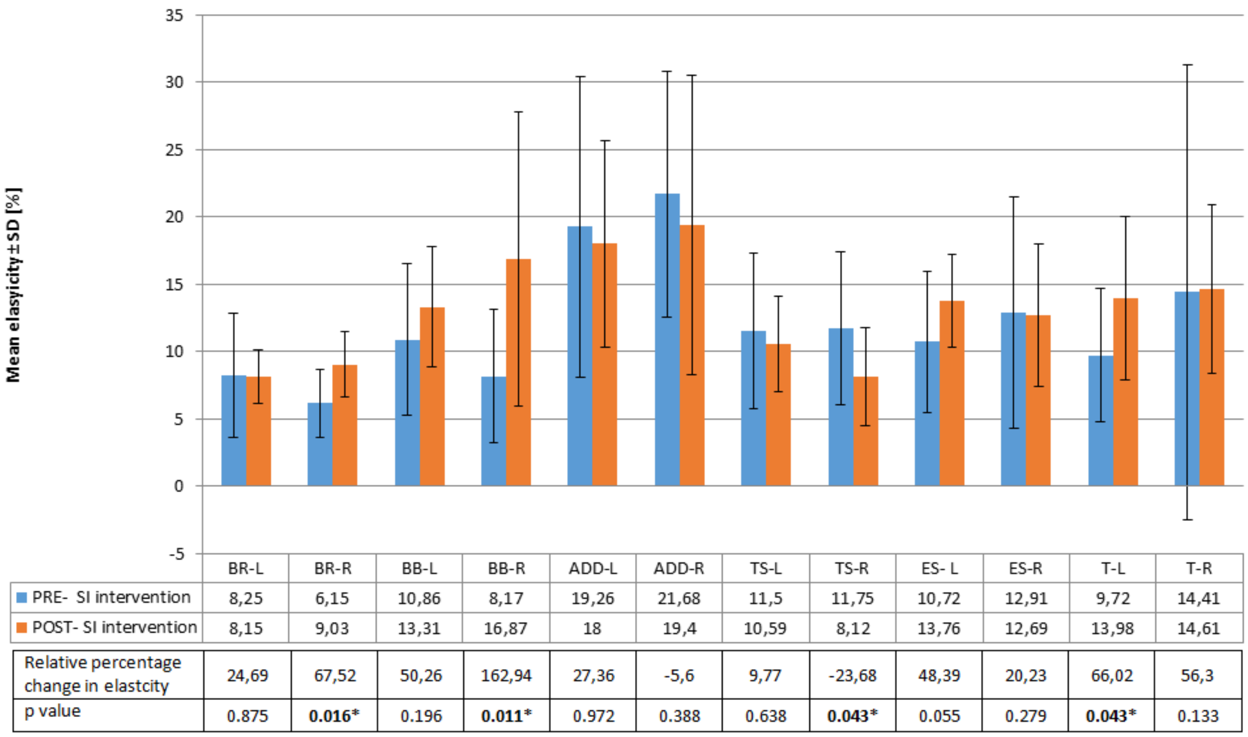

	BR-L	BR-R	BB-L	BB-R	ADD-L	ADD-R	TS-L	TS-R	ES- L	ES-R	T-L	T-R
PRE- SI intervention	8,25	6,15	10,86	8,17	19,26	21,68	11,5	11,75	10,72	12,91	9,72	14,41
POST- SI intervention	8,15	9,03	13,31	16,87	18	19,4	10,59	8,12	13,76	12,69	13,98	14,61
Relative percentage change in elastcity	24,69	67,52	50,26	162,94	27,36	-5,6	9,77	-23,68	48,39	20,23	66,02	56,3
p value	0.875	**0.016***	0.196	**0.011***	0.972	0.388	0.638	**0.043***	0.055	0.279	**0.043***	0.133

Figure 5. Mean change in FT elasticity pre and post SI intervention.
